# Supplementary material for: Transcription readthrough is prevalent in healthy human tissues and associated with inherent genomic features
Source: Commun Biol. 2024 Jan 15;7:100. doi: 10.1038/s42003-024-05779-5 (PMC10789751; doi:10.1038/s42003-024-05779-5)
Supplement: Supplementary file 2 — Supplementary Information [file 42003_2024_5779_MOESM2_ESM.pdf]

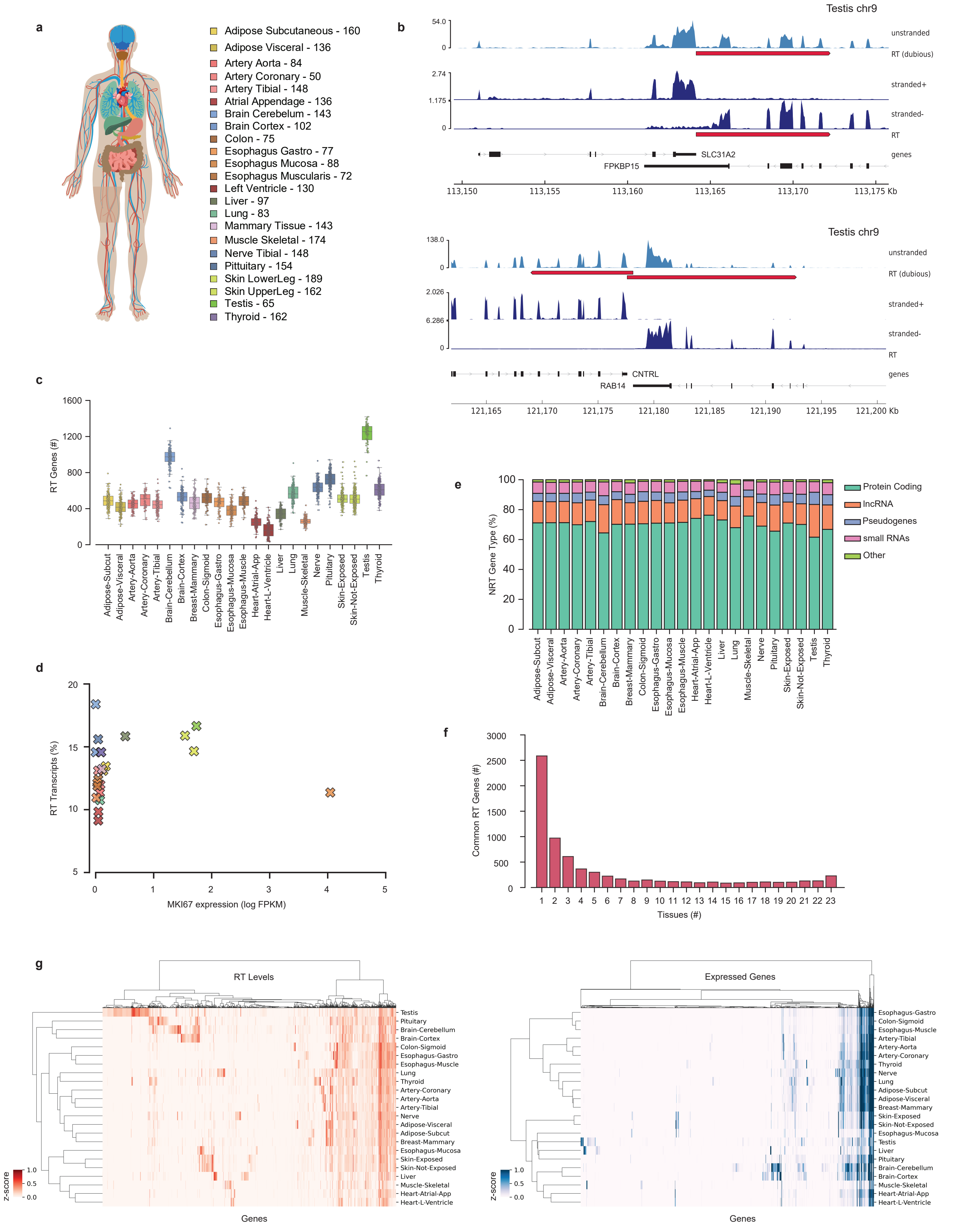

Supplementary Figure 1: Transcription readthrough prevalence in human healthy tissues. a) Schematic representation of the assessed human healthy tissues from the GTEX project. The final number of assessed samples for each tissue is indicated. Human illustration design by Vexels. **b)** RNAseq profiles for genes SLC31A2, CNTRL and RAB14 showing dubious cases of transcription readthrough region (red) in skeletal muscle using unstranded and strand-specific RNAseq libraries. RNAseq coverage is represented as reads per kilobase million (RPKMs). Boxes represent exons, separated by introns shown as solid lines. Assembly GRCh38, gencode annotation v37. **c)** Number of RT genes detected for each sample across the different tissues. Boxplot whiskers represent the 25th and 75th percentiles, while the mid-line represents the median. **d)** Scatterplot of RT transcripts percentage relative to all expressed genes and MKI67 expression levels (log2 FPKMs) in the respective tissue. **e)** Proportion of NRT genes detected for each tissue grouped by gene type: protein-coding, lncRNA, pseudogenes and other genes. **f)** Number of common RT genes in clusters of tissues. **g)** Clustering analysis of RT levels (RT ratio) versus all expressed genes (Methods for details).

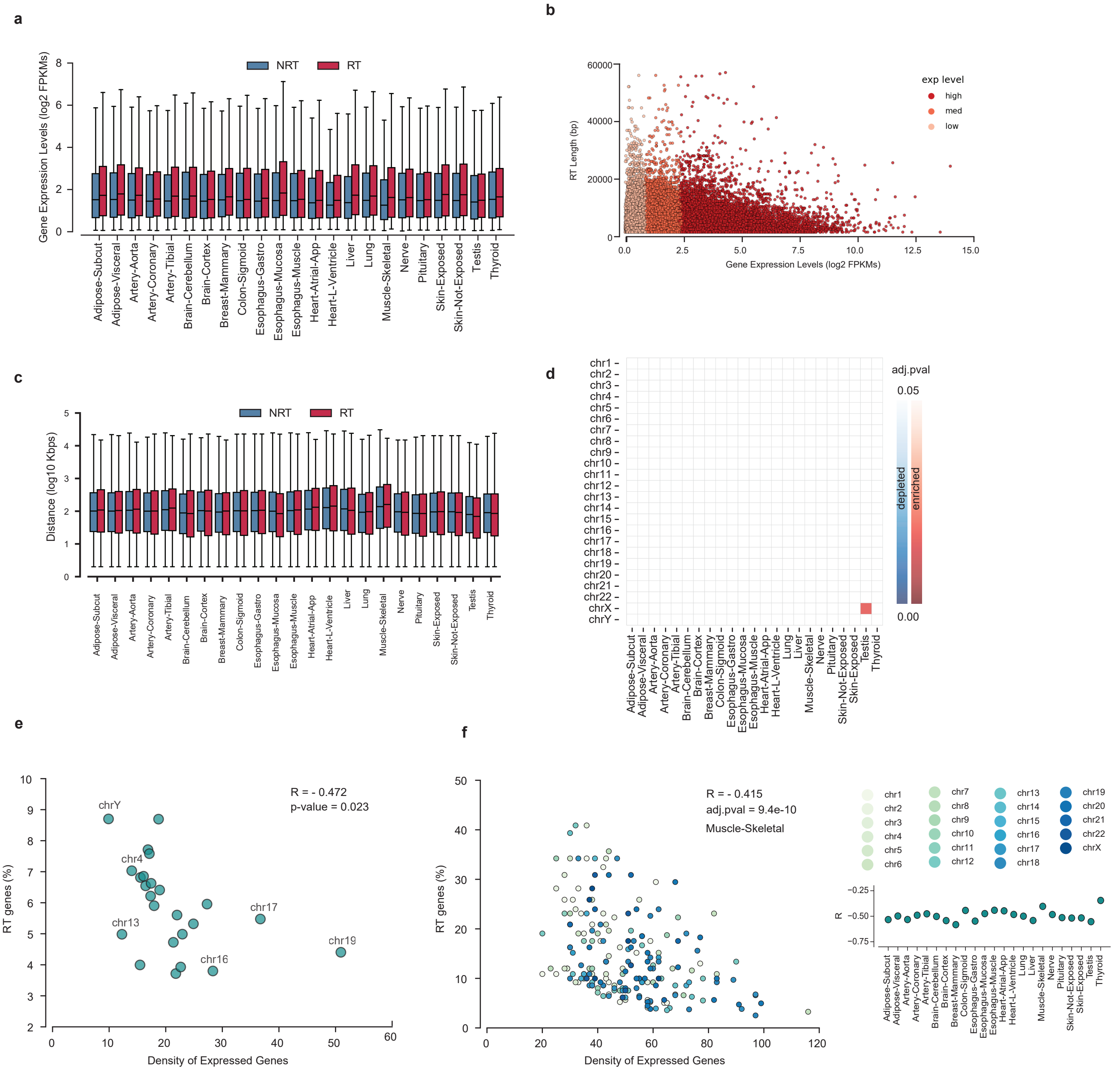

Supplementary Figure 2. Genomic features of RT genes in healthy tissues. a) Expression levels of RT and NRT genes (log2 FPKMs) for each tissue. Cohen's d<0.25 and Mann-Whitney rank test FDR < 0.05, except for the liver. Boxplot whiskers represent the 25th and 75th percentiles, while the mid-line represents the median. **b)** Relation between gene expression levels and RT length. Genes are colored by the relative level of expression within each tissue (high, medium and low). **c)** Distance between the RT and NRT genes to the nearest downstream neighbor gene in tandem (log10 Kbps). Cohen's d <0.30 and Mann-Whitney rank test FDR < 0.05. Boxplot whiskers represent the 25th and 75th percentiles, while the mid-line represents the median. **d)** Heatmap representing the enrichment (red) or depletion (blue) of NRT genes proportion across the different chromosomes for each tissue. **e)** Scatterplot of RT transcripts percentage in all tissues and density of expressed genes for each chromosome in RNAseq stranded data. **f)** Scatterplot of RT transcripts percentage and density of expressed genes for each chromosome region of 1 mega base pairs in muscle skeletal tissue (left plot). Spearman correlation coefficients for all tissues with FDR < 0.05 (right plot).

a

#	MNEMONIC	DESCRIPTION	H3K9me3	H3K36m3	H420me1	H3K79me2	H3K4me1	H3K27ac	DNase	H3K9ac	H3K4me3	H3K4m2	H2A.Z	H3K27me3	%STATE
1.0	TssA	Active TSS	0.4	0.1	0.0	5.0	0.4	89.3	92.3	96.5	99.9	99.1	86.4	3.5	0.18
2.0	PromU	Promoter Upstream TSS	0.6	0.0	0.5	6.2	99.3	91.5	82.4	96.1	99.6	100.0	95.6	23.4	0.40
3.0	PromD1	Promoter Downstream TSS 1	0.2	1.7	7.0	98.4	60.8	99.8	92.2	100.0	100.0	100.0	93.2	6.3	0.41
4.0	PromD2	Promoter Downstream TSS 2	0.9	7.3	20.3	94.0	87.5	52.3	8.0	55.1	86.7	98.0	7.2	5.2	0.18
5.0	Tx5	Transcribed - 5' preferential	0.4	1.1	17.0	76.2	0.3	0.2	0.7	0.0	0.0	0.1	0.0	0.1	2.21
6.0	Tx	Strong transcription	0.7	94.2	45.7	80.9	7.2	1.0	1.0	0.0	0.0	0.4	0.0	0.1	0.69
7.0	Tx3	Transcribed - 3' preferential	0.1	85.8	1.3	0.8	0.1	0.0	0.5	0.0	0.0	0.0	0.0	0.0	3.46
8.0	TxWk	Weak transcription	0.0	2.4	0.1	1.3	0.1	0.0	0.5	0.0	0.0	0.0	0.0	0.0	5.95
9.0	TxReg	Transcribed & regulatory (Prom/Enh)	0.2	27.5	60.2	98.1	98.3	99.9	72.1	92.8	74.0	99.6	6.1	1.3	0.31
10.0	TxEnh5	Transcribed 5' preferential and Enh	0.2	25.9	49.5	96.2	94.1	94.6	25.7	5.8	2.0	41.6	5.8	0.2	0.38
11.0	TxEnh3	Transcribed 3' preferential and Enh	0.4	89.6	14.8	11.0	74.3	50.0	20.3	2.9	1.4	11.8	2.9	0.5	0.21
12.0	TxEnhW	Transcribed and Weak Enhancer	0.1	9.3	48.3	95.5	76.8	3.2	6.6	0.0	0.4	18.9	0.0	0.8	0.51
13.0	EnhA1	Active Enhancer 1	0.2	4.0	1.3	5.9	99.3	99.9	83.7	95.7	38.3	95.7	43.3	0.4	0.24
14.0	EnhA2	Active Enhancer 2	0.2	0.5	0.8	2.6	97.4	97.2	59.1	7.5	9.6	96.8	7.5	0.6	0.33
15.0	EnhAF	Active Enhancer Flank	0.3	0.4	0.5	2.1	97.7	94.5	31.0	3.7	1.2	2.3	3.7	0.7	0.49
16.0	EnhW1	Weak Enhancer 1	0.1	0.0	0.2	0.5	91.2	16.8	39.9	3.4	15.2	39.1	3.4	0.9	0.27
17.0	EnhW2	Weak Enhancer 2	0.1	0.2	0.5	1.0	75.9	0.4	13.8	0.0	0.0	1.3	0.0	0.5	0.90
18.0	EnhAc	Primary H3K27ac possible Enhancer	0.3	0.3	0.1	1.1	4.9	64.3	19.4	0.7	0.5	3.3	0.7	0.4	0.26
19.0	DNase	Primary DNase	0.1	0.0	0.1	0.0	3.4	0.3	44.7	0.0	0.0	1.4	0.0	0.1	0.60
20.0	ZNF/Rpts	ZNF genes & repeats	88.9	82.0	1.0	15.9	0.5	0.1	0.6	0.2	4.7	1.4	0.2	0.1	0.16
21.0	Het	Heterochromatin	69.6	0.2	0.0	0.0	0.1	0.0	1.4	0.0	0.2	0.1	0.0	0.5	0.84
22.0	PromP	Poised Promoter	2.6	0.3	0.2	2.0	9.6	11.0	19.6	9.1	34.5	67.6	9.1	1.0	0.20
23.0	PromBiv	Bivalent Promoter	2.2	0.3	2.4	4.0	76.6	15.6	29.5	23.9	63.9	83.1	23.9	96.6	0.24
24.0	ReprPC	Repressed Polycomb	1.1	0.1	0.3	0.3	3.4	0.2	1.2	0.0	0.1	0.3	0.0	72.4	1.27
25.0	Quies	Quiescent/Low	0.1	0.0	0.0	0.0	0.0	0.0	0.0	0.0	0.0	0.0	0.0	0.1	78.60

b

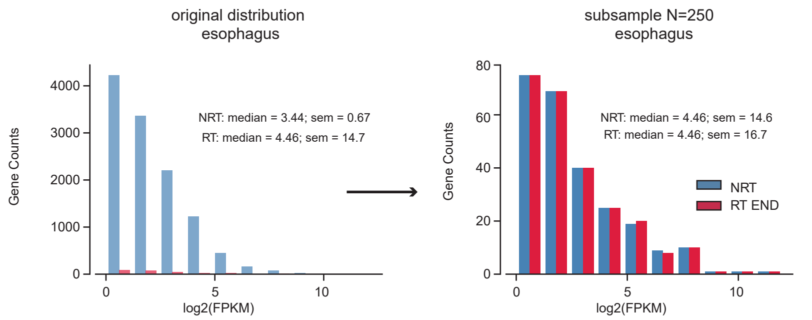

c

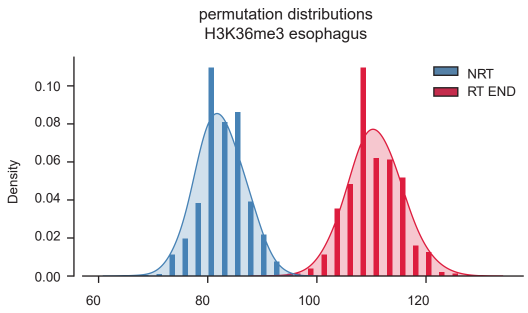

d

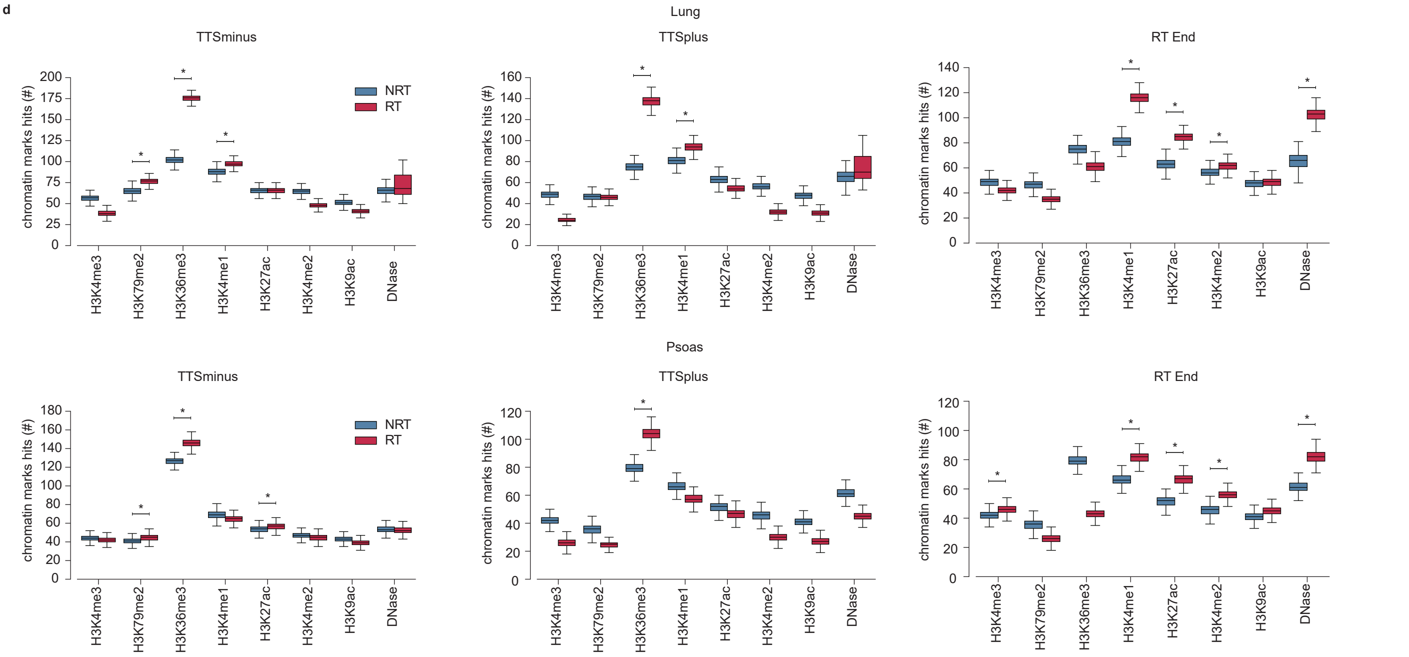

Supplementary Figure 3. a) Dictionary of chromatin states. **b**) As RT and NRT groups differ considerably in size and expression, 1000 equal-sized expression-matched subsamples were built by randomly selecting subsets of RT genes from each tissue (N=200) and finding the nearest neighbor expression partner in the group of NRT genes. Median and standar error of the mean (sem) are indicated. **c**) Permutation analysis distribution (N=1000) of the number of RT and NRT genes with the chromatin mark H3K36me3me1 in the TTSplus region of the esophagus. **d**) Number of RT/NRT genes and RT ends with chromatin marks associated with the enriched chromatin states in different regions for lung and psoas. Boxplot whiskers represent the 25th and 75th percentiles, while the mid-line in each box represents the median. The '*' indicates statistically significant differences: Mann-Whitney test p-value < 0.01; Cohen's d > 1.

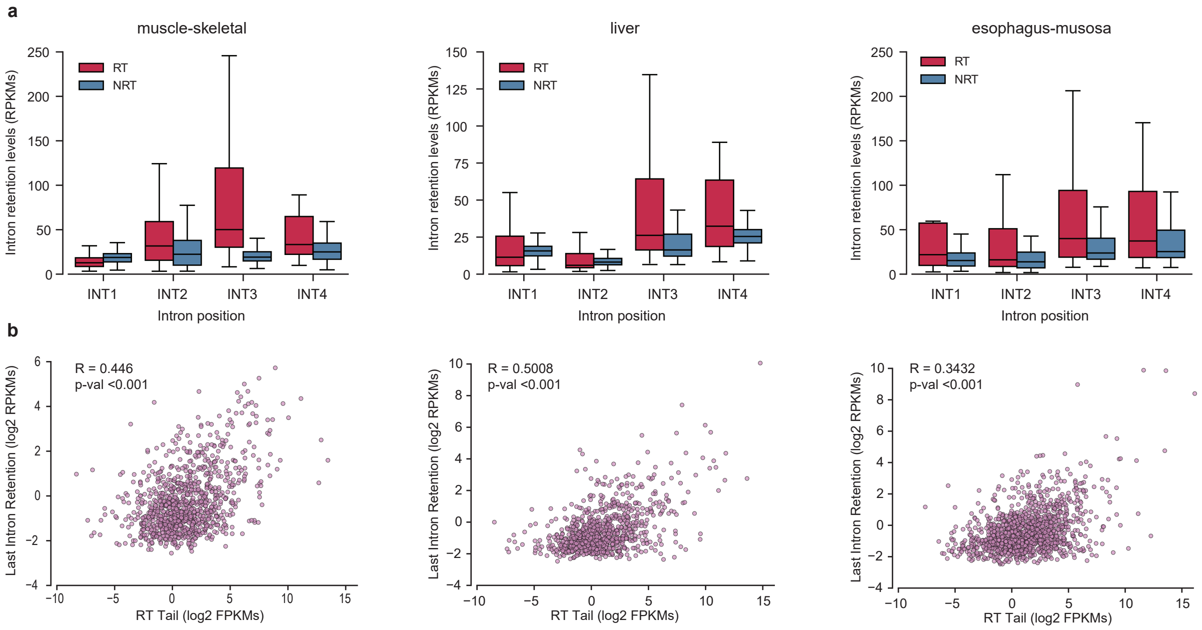

Supplementary Figure 4. Intron retention and Transcription readthrough in healthy tissues. a) Intron retention levels in first, second, second -last and last introns of RT and NRT genes for skeletal muscle (left), liver (center) and brain-cerebellum (right). Boxplot whiskers represent the 25th and 75th percentiles, while the mid-line represents the median. All differences are significant: Mann-Whitney test p-value < 0.05 and Cohen's d > 0.3. **b)** Scatterplot of expression levels of the RT region and respective intron retention levels of the last intron for the tissues in A (log2 FPKMs). Correlation coefficient is given by Spearman.

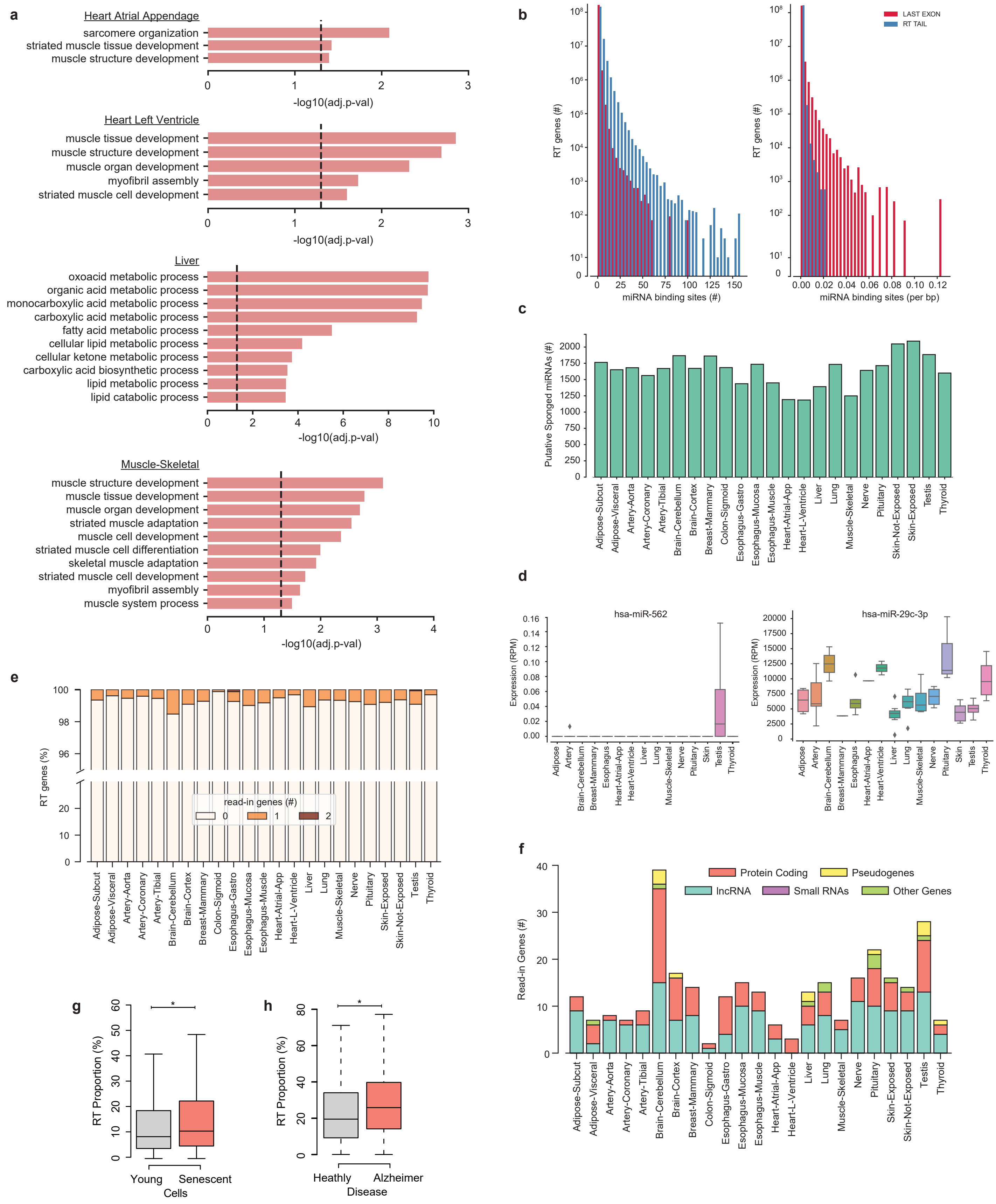

Supplementary Figure 5. Cellular impact of Transcription readthrough. a) Top biological processes significantly enriched in RT genes in several human tissues (-log10 adj. p-value). **b)** miRNA binding sites in the last exon and tail of RT genes in absolute number (left figure) and normalized by region length (right figure). **c)** Number of miRNAs with RT transcripts acting as putative sponges. **d)** miRNAs expression levels for tissue-specific putative sponged miRNAs. E) Proportion of RT transcripts reaching downstream genes. **f)** Proportion of read-in genes detected for each tissue grouped by gene type: protein-coding, lncRNA, pseudogenes and other genes. **g)** Readthrough Proportion of RT genes in replicative and senescent human cells. **h)** Readthrough Proportion of RT genes in the cortical regions from healthy individuals and Alzheimer's patients. *Mann-Whitney test p-value < 0.05. Boxplot whiskers represent the 25th and 75th percentiles, while the mid-line represents the median.
