# Supplementary material for: Transcription readthrough is prevalent in healthy human tissues and associated with inherent genomic features
Source: Commun Biol. 2024 Jan 15;7:100. doi: 10.1038/s42003-024-05779-5 (PMC10789751; doi:10.1038/s42003-024-05779-5)
Supplement: Supplementary file 3 — Description of Additional Supplementary Files [file 42003_2024_5779_MOESM3_ESM.pdf]

Description of Additional Supplementary Files

File name: Supplementary Data 1
Description: RT genes detect for each tissue referring: the number of samples, expressed genes, RT genes.

File name: Supplementary Data 2
Description: Correlation between RT genes and technical attributes of GTEx samples.

File name: Supplementary Data 3
Description: Percentage of RT genes and density of expressed genes across the chromosomes.

File name: Supplementary Data 4
Description: Percentage of RT genes for each intron number class.

File name: Supplementary Data 5
Description: Hexamer enrichment results for the 500 bp after the termination site of RT and NRT genes of each tissue.

File name: Supplementary Data 6
Description: miRNA sponges detected across healthy tissues.
